# Supplementary material for: RNA Modification Level Estimation with pulseR
Source: Genes (Basel). 2018 Dec 10;9(12):619. doi: 10.3390/genes9120619 (PMC6316556; doi:10.3390/genes9120619)
Supplement: Supplementary file 1 [file genes-09-00619-s001.tgz › supplement/supplementary_figures.pdf]

RNA modification level estimation with pulseR: Supplementary figures

Etienne Boileau, Christoph Dieterich

December 2, 2018

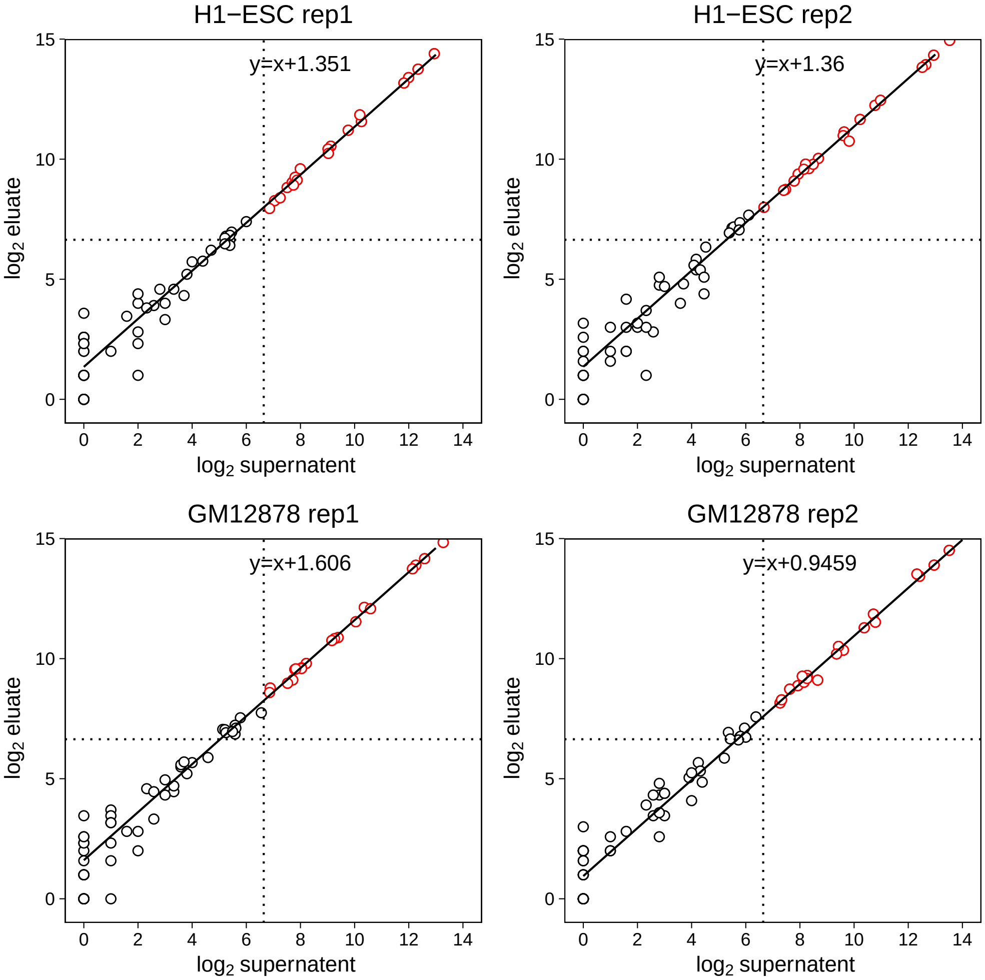

Figure 1: Linear regression of ERCC read pairs for supernatant and eluate for all samples. Red circles represent ERCC RNAs that were used for the fitting. Dotted lines indicate the threshold of 100 read counts used for filtering low-concentration RNAs. The solid line indicate the regression line. Regression function is indicated at the top of each figure. All fits are significant.

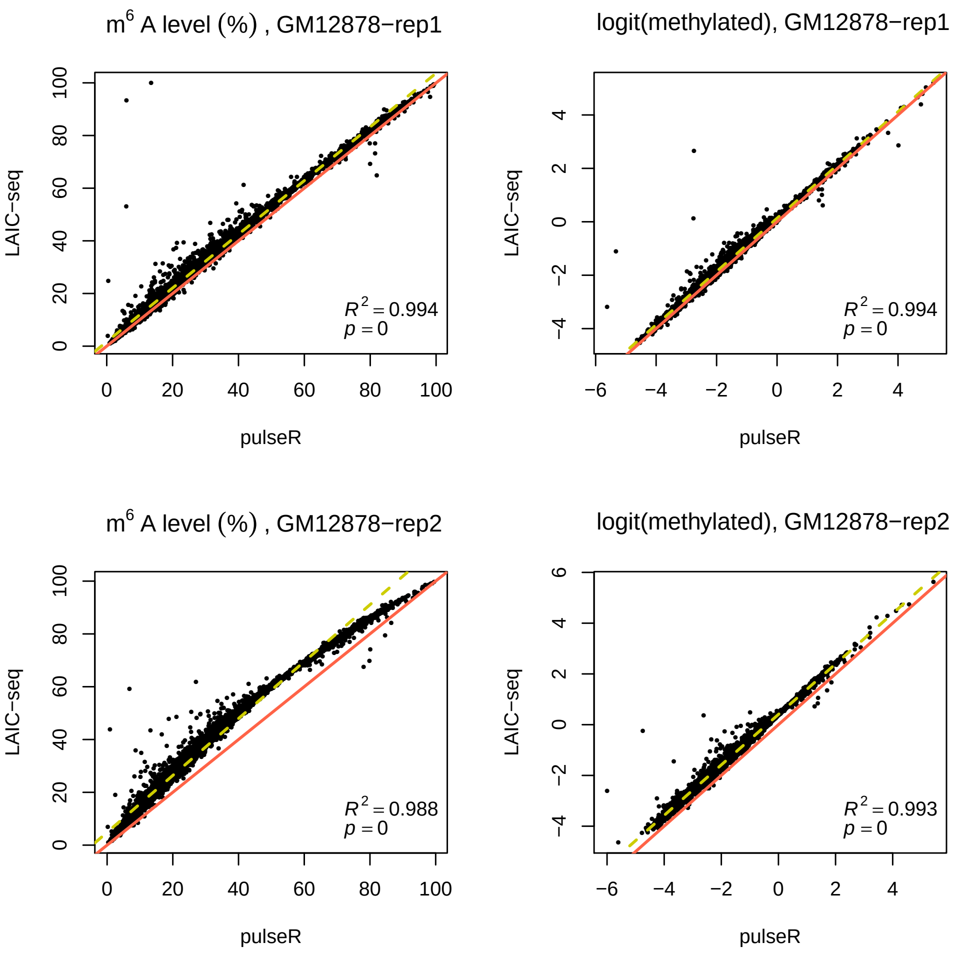

Figure 2: Comparison of m^6A methylation levels between pulseR estimates and the LAIC-seq method for replicates of one cell line (GM12878) without spike-ins, represented as a proportion (left) or using log-odds (right). Line of equality in red, linear fit in yellow with coefficient of determination.

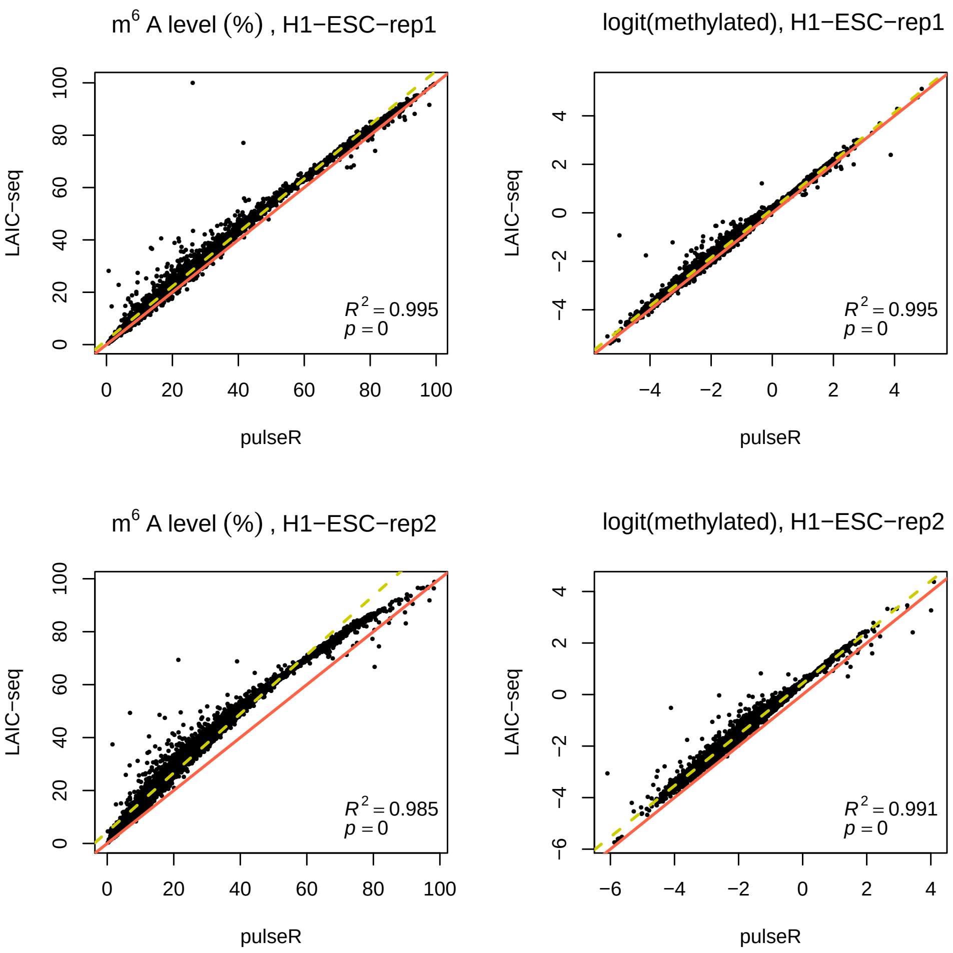

Figure 3: Comparison of m^{6}A methylation levels between pulseR estimates and the LAIC-seq method for replicates of one cell line (H1-ESC) without spike-ins, represented as a proportion (left) or using log-odds (right). Line of equality in red, linear fit in yellow with coefficient of determination.

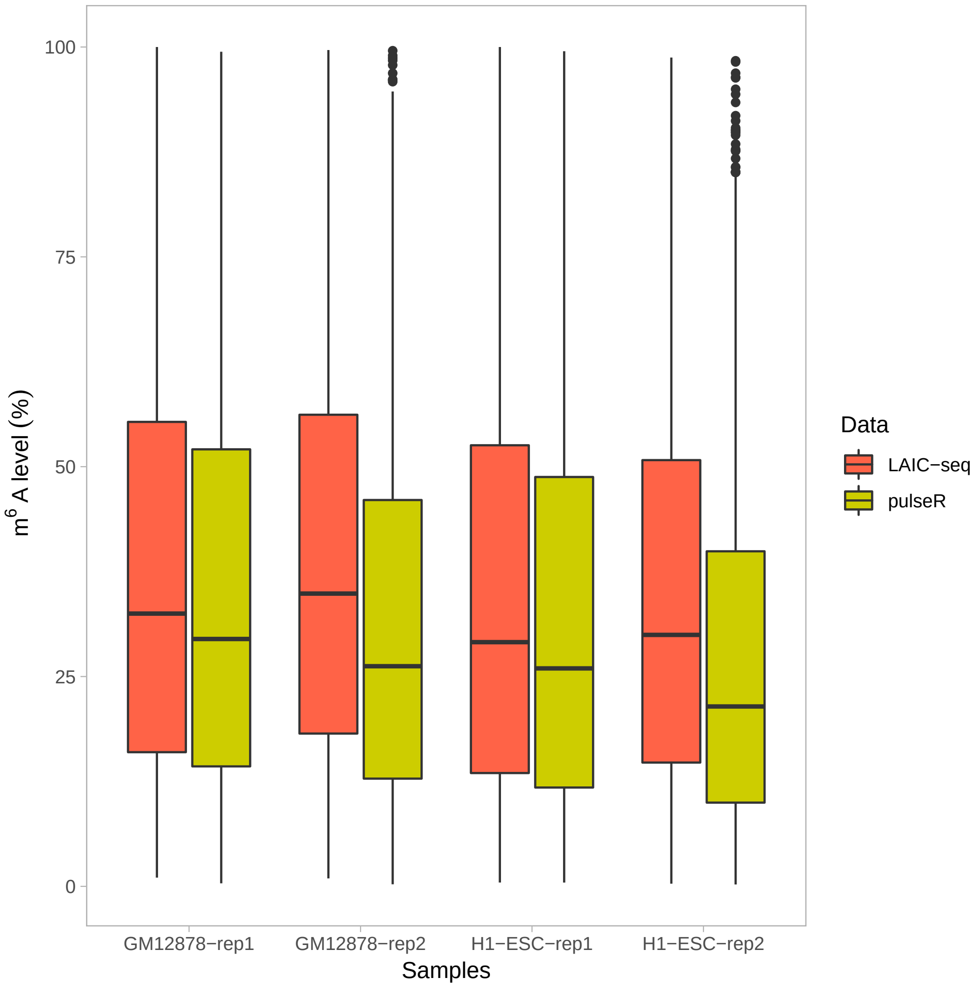

Figure 4: Sample comparisons of m^6A levels between pulseR estimates and the LAIC-seq method, when no spike-ins are used for fitting the scaling factors (p-values<2.2e-16, two-sided Wilcoxon rank sum test).

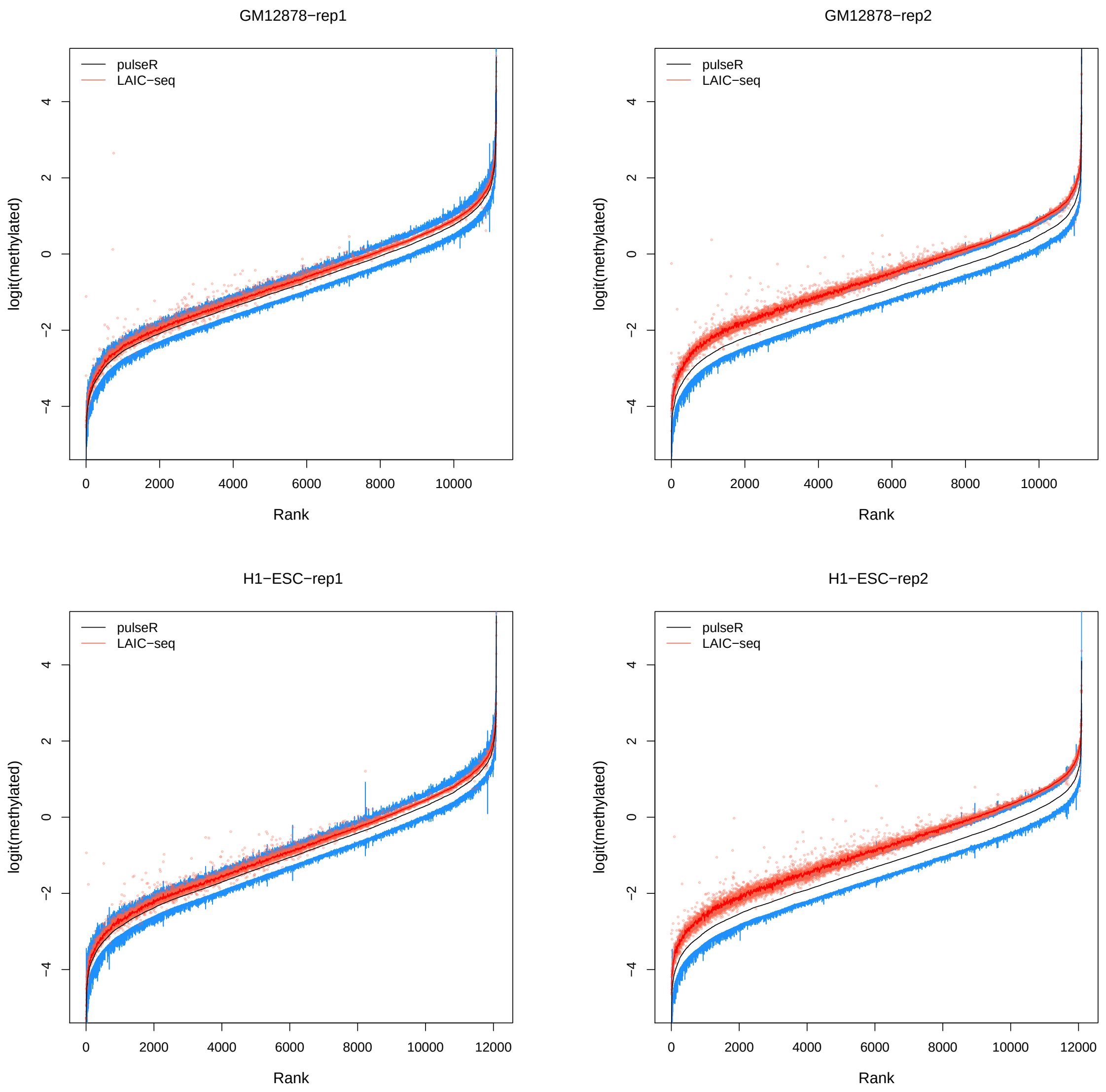

Figure 5: Confidence intervals for all samples, without spike-ins. Genes are sorted by their log-odds of methylation γ (black line). Estimates obtained with the LAIC-seq method are shown as red dots with a running median. Upper and lower 95% confidence interval boundaries are show in blue (above/below mean value in black).

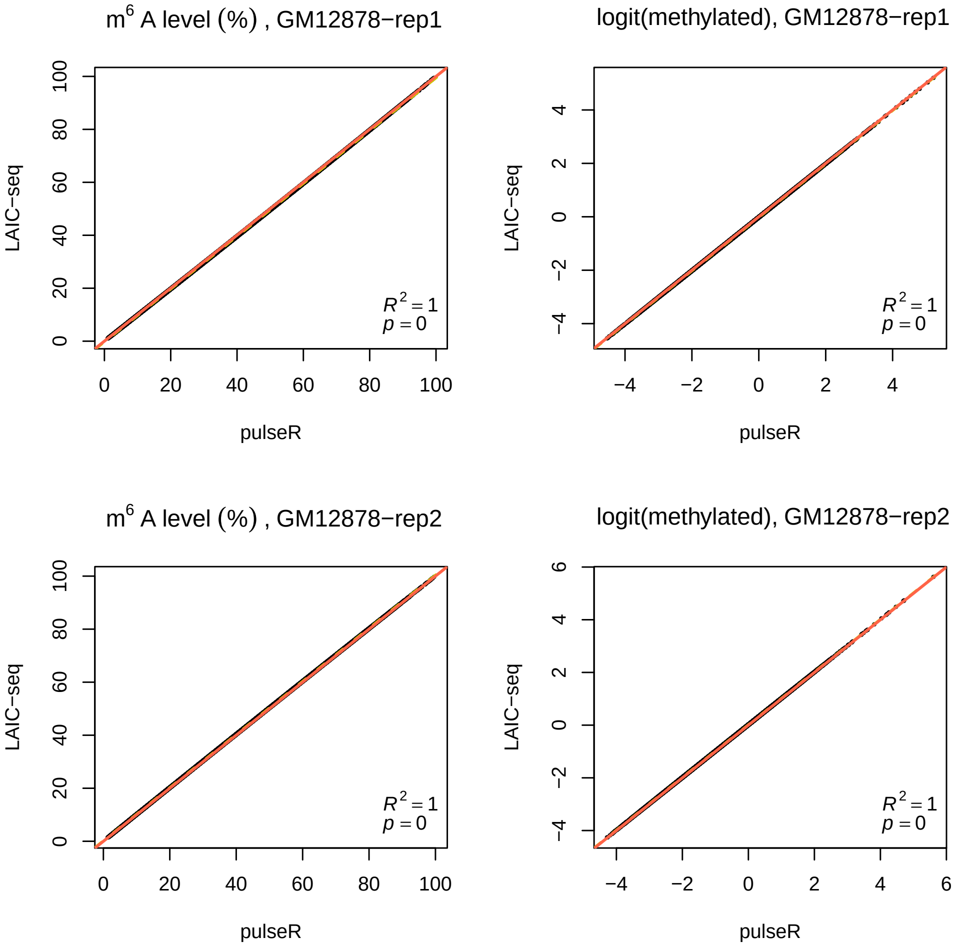

Figure 6: Comparison of m^6A methylation levels between pulseR estimates and the LAIC-seq method for replicates of one cell line (GM12878) with spike-ins, represented as a proportion (left) or using log-odds (right). Line of equality in red, linear fit in yellow with coefficient of determination.

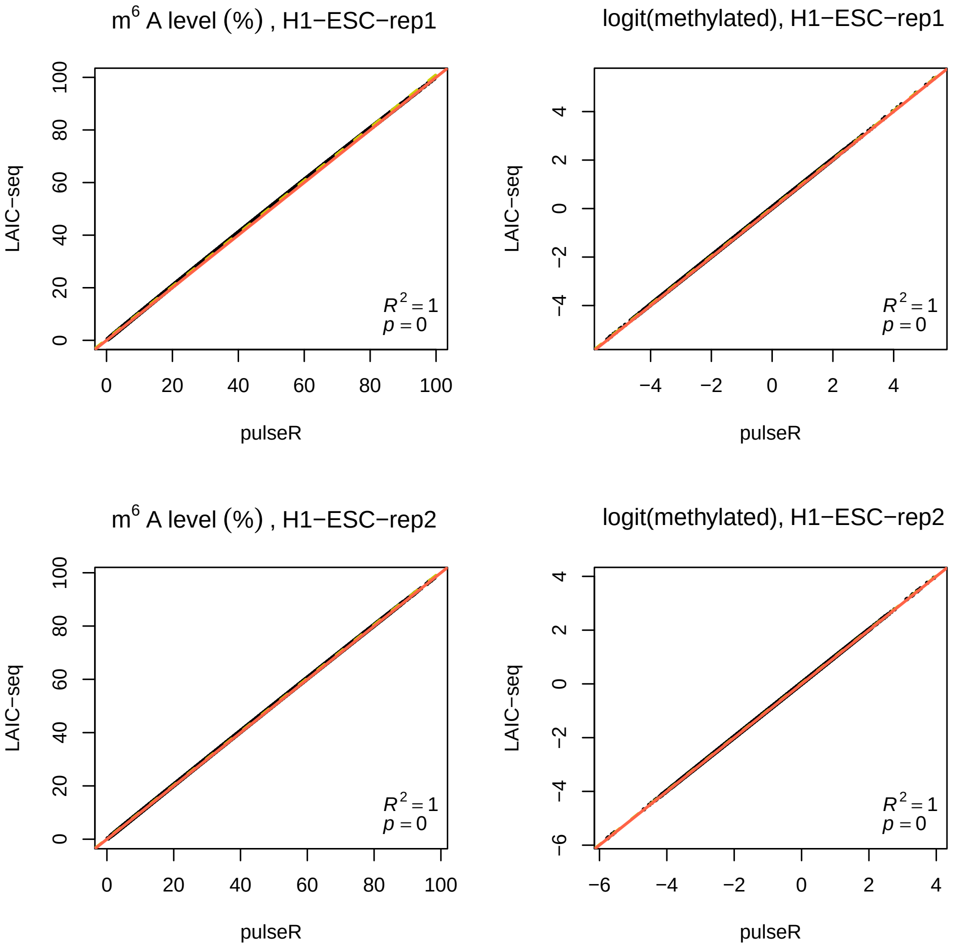

Figure 7: Comparison of m^{6}A methylation levels between pulseR estimates and the LAIC-seq method for replicates of one cell line (H1-ESC) with spike-ins, represented as a proportion (left) or using log-odds (right). Line of equality in red, linear fit in yellow with coefficient of determination.

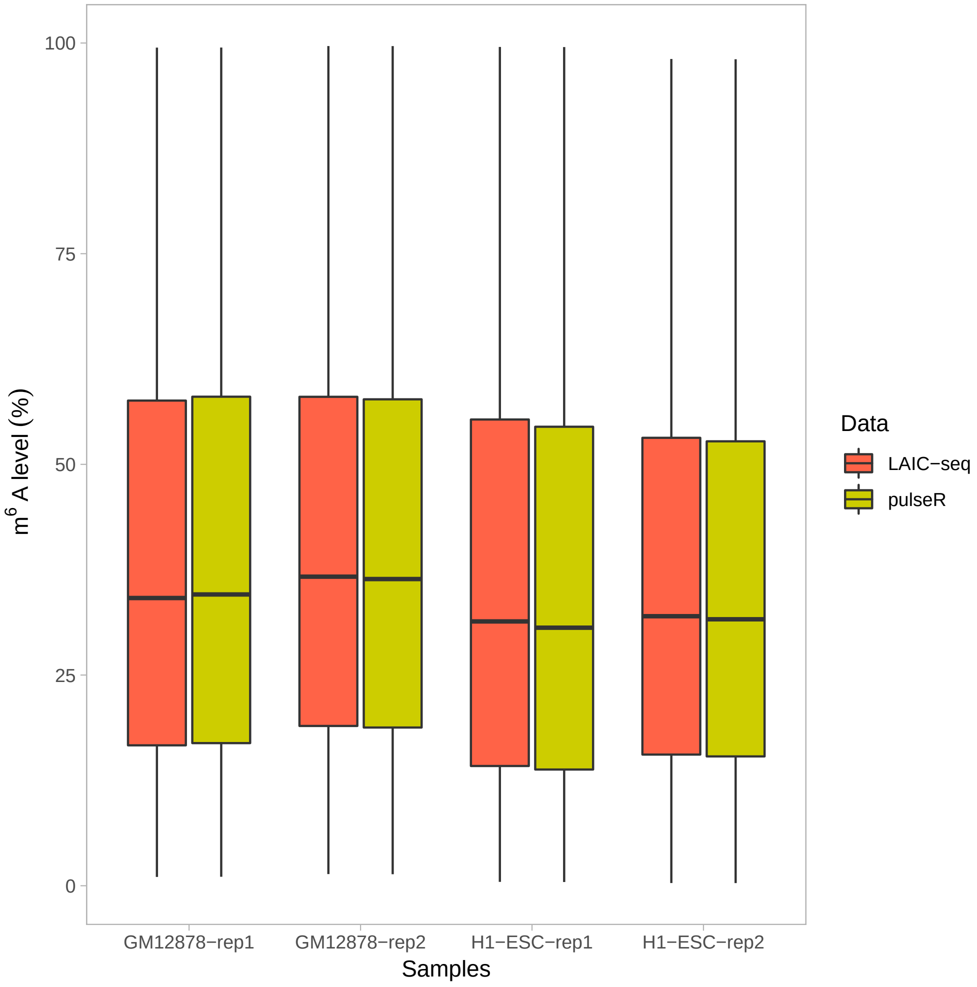

Figure 8: Sample comparisons of m^6A levels between pulseR estimates and the LAIC-seq method, when spike-ins are used for fitting the scaling factors (differences are not statistically significant at α=0.05, two-sided Wilcoxon rank sum test).

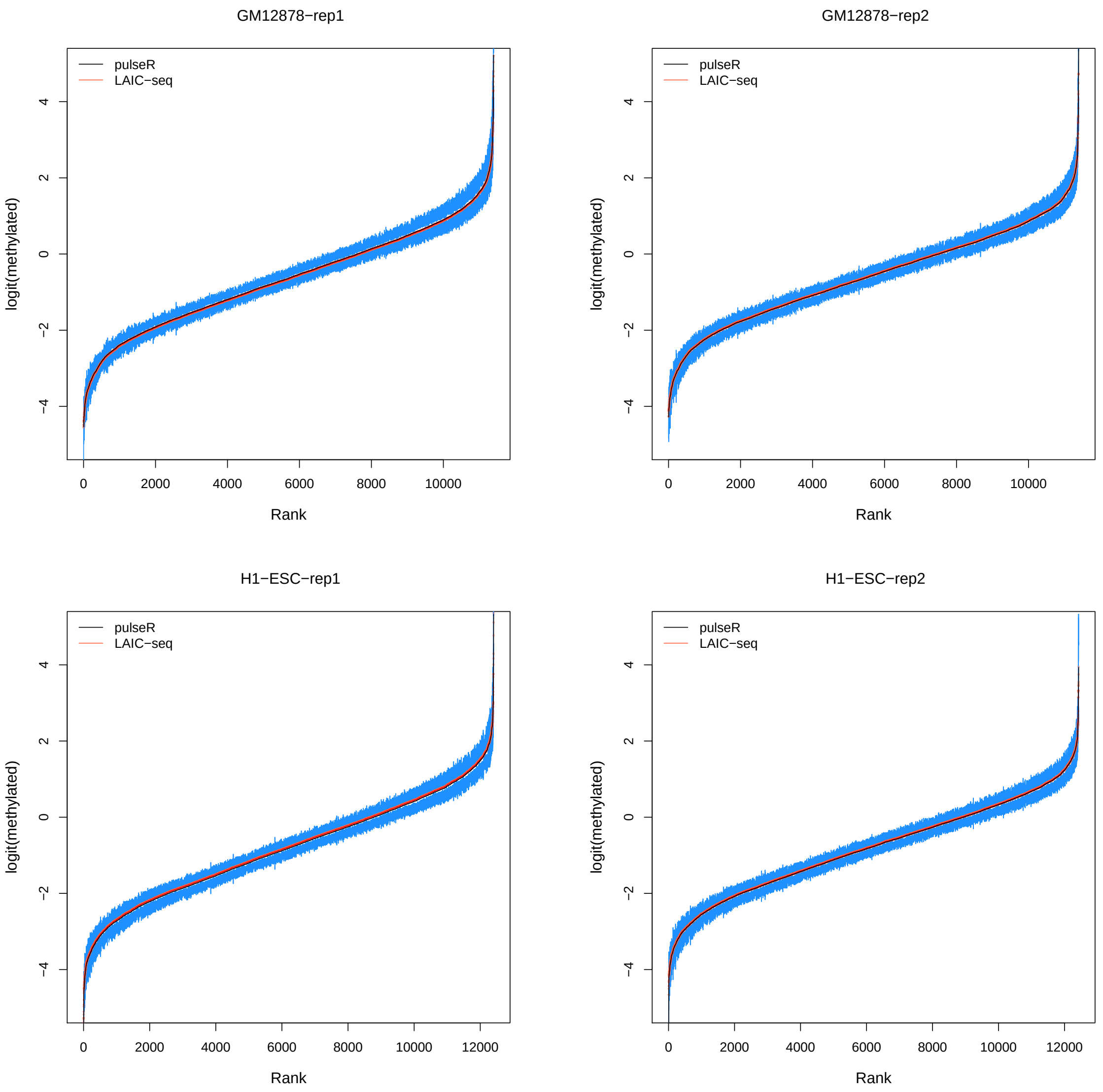

Figure 9: Confidence intervals for all samples, with spike-ins. Genes are sorted by their log-odds of methylation γ (black line). Estimates obtained with the LAIC-seq method are shown as red dots with a running median. Upper and lower 95% confidence interval boundaries are show in blue (above/below mean value in black).
